# Cell-type specific changes in PKC-delta neurons of the central amygdala during alcohol withdrawal

**DOI:** 10.1038/s41398-022-02063-0

**Published:** 2022-07-20

**Authors:** Geoffrey A. Dilly, Cory W. Kittleman, Tony M. Kerr, Robert O. Messing, R. Dayne Mayfield

**Affiliations:** 1 Institute for Neuroscience, University of Texas at Austin, Austin, TX 78712 USA; 2 Department of Neuroscience, The University of Texas at Austin, Austin, TX 78712 USA; 3 Waggoner Center for Alcohol and Addiction Research, The University of Texas at Austin, Austin, TX 78712 USA; 4 Department of Neurology, The University of Texas at Austin, Austin, TX 78712 USA; 5 College of Pharmacy, The University of Texas at Austin, Austin, TX 78712 USA

**Keywords:** Molecular neuroscience, Addiction

## Abstract

The central amygdala (CeA) contains a diverse population of cells, including multiple subtypes of GABAergic neurons, along with glia and epithelial cells. Specific CeA cell types have been shown to affect alcohol consumption in animal models of dependence and may be involved in negative affect during alcohol withdrawal. We used single-nuclei RNA sequencing to determine cell-type specificity of differential gene expression in the CeA induced by alcohol withdrawal. Cells within the CeA were classified using unbiased clustering analyses and identified based on the expression of known marker genes. Differential gene expression analysis was performed on each identified CeA cell-type. It revealed differential gene expression in astrocytes and GABAergic neurons associated with alcohol withdrawal. GABAergic neurons were further subclassified into 13 clusters of cells. Analyzing transcriptomic responses in these subclusters revealed that alcohol exposure induced multiple differentially expressed genes in one subtype of CeA GABAergic neurons, the protein kinase C delta (PKCδ) expressing neurons. These results suggest that PKCδ neurons in the CeA may be uniquely sensitive to the effects of alcohol exposure and identify a novel population of cells in CeA associated with alcohol withdrawal.

## Introduction

The central amygdala (CeA) is a primarily GABAergic nucleus that contains multiple subtypes of inhibitory neurons [1, 2]. These GABAergic subtypes have been classified by the expression of unique marker genes, such as the peptide neurotransmitters that they release, or the various receptors and kinases that they express [1, 3, 4]. Within the CeA, these neurons form complex reciprocating microcircuits that control emotional behaviors, including stress responses, food seeking, and the consumption of addictive drugs [5–7]. CeA GABAergic subtypes can have opposing effects on these behaviors [5, 8–10], which necessitates studying the region at a cellular level.

The CeA is also a crucial mediator of pathological alcohol consumption, dependence, and withdrawal [1, 11–13]. Multiple subtypes of CeA neurons modulate alcohol drinking, including corticotropin-releasing factor (CRF, *Crh)* neurons [14], neurotensin (NTS, *Nts*) neurons [15], neurons that release dynorphin [16], and recently, neurons that express protein kinase C-δ (PKCδ) [17]. Some of these cells are specifically involved in alcohol dependence and withdrawal. CRF neuron activity only promotes alcohol consumption in alcohol-dependent animals, and also promotes somatic signs of withdrawal [14, 18]. PKCδ neurons drive alcohol consumption despite the presence of a paired electric shock, and such behavior correlates with escalated drinking and alcohol dependence [17], and stress induced alcohol seeking [1]. Additionally, alcohol can have cell-type specific effects on neurons of the central amygdala. For example, CeA-CRF neurons and PKCδ neurons have increased cFos activity during alcohol withdrawal [14, 17, 19].

Bulk transcriptomics studies have demonstrated that alcohol exposure can alter gene expression in the CeA [20–24], and it is hypothesized that these changes underlie escalated alcohol consumption in dependent animals. These studies have implicated several novel biological pathways and molecular mechanisms that may serve as targets for treatment or future research. For example, alcohol alters the expression of genes that are associated with CRF signaling, cell activation, and synaptic transmission [20, 22]. While these bulk transcriptome studies have been critical in identifying molecular targets of alcohol action, they lack the cell-type specific context required to better understand circuit-level molecular mechanisms. In addition, non-neuronal cells contribute to features of alcohol dependence. For example, microglia are required for escalation of drinking that is associated with alcohol dependence, and eliminating them perturbs GABAergic and glutamatergic gene expression in the CeA [23]. Bulk sequencing experiments have identified affected cells based on deconvolution and enrichment analyses [20]. However, direct, cell-type specific characterization of the CeA has not been reported. One study has examined single-cell transcriptional changes in rat CeA during morphine withdrawal, but used qPCR, not mRNA sequencing, and did not attempt to determine the composition of the CeA [25]. Clearly it is important to understand the cell-type specific consequences of alcohol dependence and withdrawal. Understanding alcohol’s effects on individual cell types is necessary for understanding how neural circuits in the CeA contribute to dependence.

In order to determine transcriptomic changes due to alcohol dependence, we compared cell-type specific transcriptomes from naive and alcohol-dependent rats during acute alcohol withdrawal. We utilized single-nuclei RNA sequencing (snRNA-seq), a technique that allowed us to obtain single-cell transcriptomes from frozen micropunches of rat CeA. We then used fluorescent in situ hybridization with RNAscope to confirm the identity and cell specificity of specific transcripts based on unique marker genes. Our findings identify a specific subpopulation of GABAergic neurons that are especially sensitive to withdrawal from alcohol dependence.

## Results

### Vapor exposure induces signs of alcohol withdrawal

Animals were treated with alcohol vapor for 4 weeks, as described in the methods. Vapor-exposed animals attained high blood ethanol concentrations during each of the weekly vapor sessions with blood alcohol concentrations of 266 ± 16 mg/dL during week 4 of vapor exposure. Alcohol withdrawal scores were determined after 9–10 h of withdrawal, immediately prior to euthanasia and brain extraction. Vapor-exposed animals displayed increased behavioral signs of withdrawal relative to control animals (Withdrawal scores: Vapor mean = 5.25 ± 0.75, median = 6; *n* = 4, Air mean = 0.66 ± 0.33, median = 1, *n* = 3; *p* value = 0.0286 by one-tailed Mann–Whitney test).

### Major cell types in the CeA

**Fig. 1:**
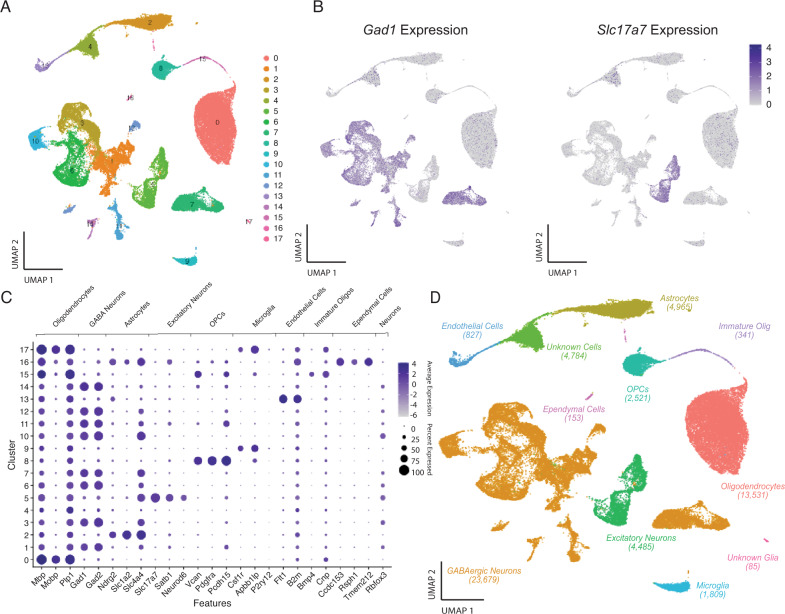
Identification of major cell types within the CeA. **A** UMAP plot of 58,640 CeA single-nuclei colored by cluster. Louvain clustering analysis (resolution = 0.1) identified 18 transcriptionally defined clusters. **B** UMAP plots showing the expression levels of select neuronal marker genes. Distinct populations of cells express *Gad1*, a marker of GABAergic inhibitory neurons, and *Slc17a7* (VGlut2), a marker of glutamatergic excitatory neurons. These data illustrate that one cluster is composed of primarily excitatory neurons, while multiple clusters are identified which represent distinct inhibitory cell classes of the CeA. **C** Major cell-type marker gene expression in CeA clusters. The identity of each cluster (0–17) was identified based on the expression of known marker genes for the major cell classes within the CeA. The size of each dot indicates the percentage of cells that contained at least one read of each marker gene; the color depth represents the log-scaled average expression, where the darkest color is the highest expressing cluster. The 24 marker genes that are displayed were identified based on previous studies of the central amygdala [26–29]. These genes were used to identify each cluster in the dataset. Two clusters (4 and 17) were not readily identifiable based on marker gene expression. **D** UMAP plot colored by the major cell types identified. Cells were classified as one of nine major cell types. Unidentified clusters comprised two additional groups, for a total of 11 types of cells in the data.

To determine the cellular identity, Louvain clustering and UMAP dimensional reduction analysis were performed based on the 2000 most variant genes across all CeA cell nuclei in the dataset. Clustering analysis (Louvain resolution = 0.1) of 58,640 nuclei identified 18 distinct clusters of transcriptomically similar nuclei (Fig. 1A). We performed marker gene analysis to determine the cell-specific identity of each cluster. Marker genes were selected based on previous studies of cell-type specific gene expression [26–29], and were not necessarily the *primary* marker of a given population (Fig. 1C). Marker gene analysis revealed that the detected clusters corresponded to major cell types in the brain (Fig. 1D*)*. It should be noted that some of the marker genes used in prior studies were not necessarily specific for cell types in the CeA. Notably, transcripts for the calcium/calmodulin-dependent protein kinase II subunit alpha gene (*Camk2a*) are specific for excitatory cells in cerebral cortex [27], yet we found that *Camk2a* was also expressed GABAergic neurons in the CeA, so we identified excitatory neurons with other markers. Ultimately, we utilized 23 genes to identify each cluster (see Methods, Fig. 1C). As expected, the pan-neuronal markers *Rbfox3* (NeuN) showed high expression in both the inhibitory and excitatory neuron clusters (Fig. 1C).

Clusters of cells identified by Louvain clustering analysis corresponded to the major cell types of the brain including inhibitory neurons, excitatory neurons, oligodendrocytes, astrocytes, and microglia (Fig. 1). Multiple large clusters were identified as glial cells. Oligodendrocytes formed the largest single cluster of cells (13,531 cells, 23%) and were identified by high expression of the myelin-associated genes *Mbp*, *Mobp*, and *Plp1* (Fig. 1C). Although *Mbp* and *Plp1* are expressed in multiple cell types, two clusters (0 and 17) had substantially higher expression of all three markers. Astrocytic markers (*Ndrg2*, *Slc1a2*, *Slc4a4*) were highly enriched in two clusters of cells (2 and 16). A cluster of microglia expressing *Csfr1*, *Apbb1lp*, and *Py2r2* was detected. Oligodendrocyte precursor cells (OPCs), immature oligodendrocytes, and epithelial cells formed smaller clusters (Fig. 1C). One cluster of putative cells (Cluster 4) was not identifiable and had very low numbers of detected genes and unique molecular identifiers (UMIs), indicating damaged cells (4784 cells, 8.2%). Small clusters of endothelial cells, ependymal cells, immature oligodendrocytes, and nonspecific glia were also identified.

GABAergic neurons were identified based on the expression of *Gad1* and *Gad2* and interestingly formed eight clusters (clusters 1, 3, 6, 7, 10, 11, 12 and 14; Fig. 1A). These neurons were the most common cell-type detected comprising 23,579 out of 58,640 cells (40.2%), which is consistent with the known anatomy of the CeA [1, 30]. A cluster of excitatory neurons (4485 cells, 7.6%) were also detected based on the markers *Neurod6, Slc17a7 and Satb1* (Fig. 1A, C). Because the CeA is largely composed of GABAergic cells, it is likely that these neurons are incidental cells from the neighboring basolateral amygdala (BLA) (Fig. 1A, C, D) [31].

### Differential gene expression in CeA astrocytes and neurons during alcohol withdrawal

**Fig. 2:**
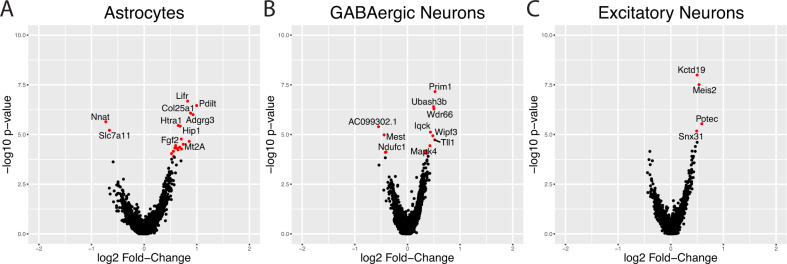
Alcohol withdrawal is associated with differential gene expression in astrocytes and neurons in the CeA. Volcano plots are shown for the three cell types with the greatest numbers of differentially expressed genes (Astrocytes **A**, GABAergic Neurons **B**, and Excitatory Neurons **C**). Genes in red indicate significant differential expression based on adjusted *p* values of <0.05, and a log2 FC of >±0.25. The top ten differentially expressed genes are labeled.

Differential gene expression analysis was used to determine cell-type specific changes in the transcriptome during alcohol withdrawal. We used a “pseudobulking” approach [27, 32, 33] for a conservative estimate of differentially expressed genes (DEGs). Counts from each animal were pooled, to create a ‘pseudobulk’ sample that could be analyzed with traditional bulk RNA tools. For our initial analysis, we collapsed the clusters based on major cell-type (shown in Fig. 1D). Differential expression analysis of the pseudobulked counts revealed transcriptional responses (adjusted *p* value < 0.05, log2 fold change > ±0.25) to alcohol withdrawal in 3 types of CeA cells (Fig. 2A, B*)*: GABAergic neurons (15 genes; 11 upregulated, 4 downregulated) (Fig. 2A), astrocytes (22 genes; 20 upregulated, 2 downregulated) (Fig. 2B), and excitatory neurons (4 upregulated genes). Volcano plots are shown in Fig. 2 to illustrate the direction and magnitude of the expression changes.

We used gene ontology and pathway analysis to identify biological processes and pathways that were perturbed in these cells during alcohol withdrawal. To facilitate pathway analysis we used a larger set of differentially expressed transcripts than we previously identified (adjusted *p* value < 0.25, log2 fold change > 0.25, 48 genes for GABAergic neurons, 38 genes for astrocytes, 9 genes for excitatory neurons). NCAS Bioplanet pathway analysis identified 9 signaling pathways that were overrepresented among the GABAergic neuron DEGs, but none in the excitatory neurons or astrocytes. Most of these pathways were associated with neuronal signaling mechanisms. Five modules were associated with nerve growth factor signaling, which indicated that nerve growth factor (NGF), fibroblast growth factor (FGF), and epithelial growth factor (EGF) signaling may be altered in GABAergic neurons during alcohol withdrawal. Modules representing other signaling pathways included GPCR signaling, adrenergic signaling, and the activation of NMDA receptors.

## Identification of GABAergic subtypes within the CeA

**Fig. 3:**
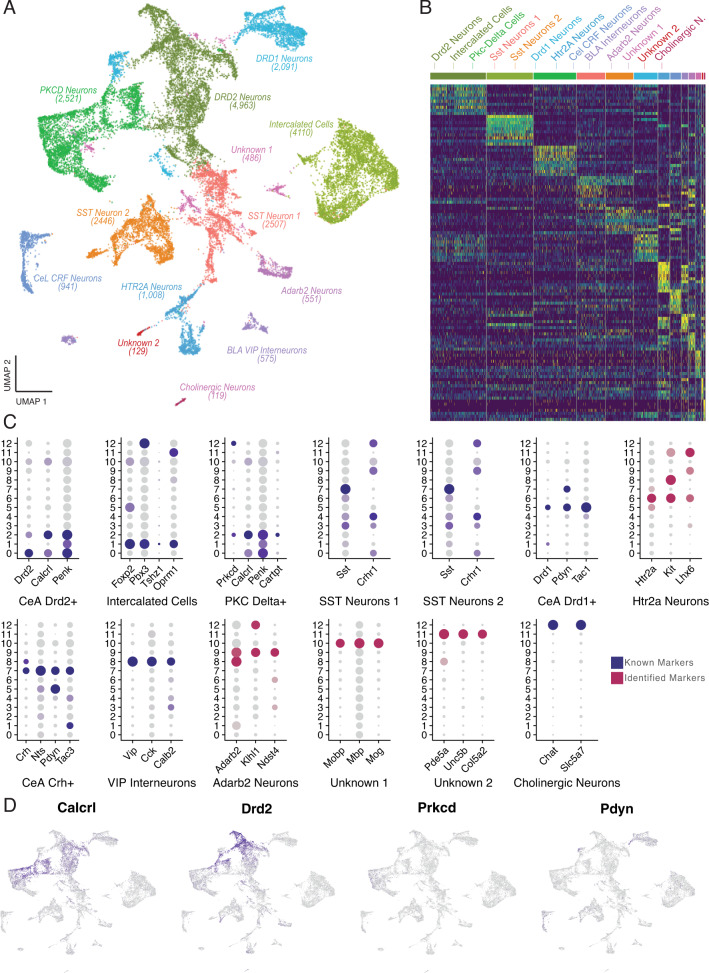
Clustering and marker gene analysis of GABAergic neurons. **A** UMAP plot of 18 clusters of putative GABAergic neurons in the CeA, colored by cluster. 15 clusters were identified as amygdalar GABAergic cells. Two clusters were not readily identifiable as GABAergic cells and contained high levels of glial genes (Unknown 1, Unknown 2). One cluster was identified as cholinergic interneurons. **B** Heatmap plot of the expression level for the top 10 marker genes in each cluster of putative GABAergic neurons. Marker gene expression in each cluster is distinct, indicating that the clustering analysis effectively identified distinct cell types of interneurons in the CeA (see Table S2 data for the list of genes plotted). **C** Marker gene analysis. Most of the clusters distinctly expressed genes that are associated with known GABAergic subtypes of CeA cells (navy blue dots). Magenta dots indicate cell types that were not identified based on known CeA gene expression, but were identified based on genes that were revealed de novo by clustering and marker gene analysis. The size of each dot indicates the percentage of cells that contained at least one read of each marker gene; the color depth represents the log-scaled average expression, where the darkest color is the highest expressing cluster. **D** UMAP plots colored by the expression of known CeA GABAergic neuron marker genes (*Calcrl, Prkcd, Drd2, Pdyn*).

As outlined above, we identified a large population of GABAergic neurons, containing multiple distinct clusters of cells. We subclustered these neurons to identify subtype specific transcriptomic changes in GABAergic neurons that were associated with alcohol withdrawal. Louvain clustering identified 13 distinct subclusters of CeA GABAergic neurons enriched with gene markers for dopamine receptors D1 and D2 (*Drd1*, *Drd2*), PKCδ (*Prkcd*), and corticotropin-releasing factor, among others (Fig. 3A). By examining DEGs between these clusters, we were able to validate that this clustering had effectively identified distinct populations of putative GABAergic neurons (Fig. 3B). Figure 3C shows the specific genes used to identify each of the subclusters. These known marker genes were distinctly expressed in specific clusters in the data. For example, the expression of selected marker genes (*Calcrl*, *Pdyn*, *Drd2*, *Prkcd*) was cluster specific on the resulting UMAP plot (Fig. 3D). Some GABAergic clusters could be identified as known subtypes of GABAergic cells, while others did not clearly match any known CeA subtypes. The latter groups were identified based on their highly expressed genes (Fig. 3B, C) [1, 4, 34–38].

The largest cluster of GABAergic neurons (cluster 0) corresponded to DRD2-expressing CeA neurons, expressing the marker genes *Drd2, Calcrl*, and *Penk*. Another cluster (cluster 5) had distinctly high expression of *Drd1, Pdyn, and Tac1*, and were identified as DRD1-expressing neurons of the CeA. Interestingly, another population (cluster 2) expressed the marker genes *Calcrl* and *Penk*, but had relatively low expression of *Drd2*, and relatively high expression of *Prkcd and Cartpt*. Cells in this cluster were identified as putative PKCδ-expressing CeA neurons (Fig. 3C, D). Another cluster (cluster 7) expressed several neuropeptide genes that are known to be localized to the lateral CeA (CeL) [36, 38]. This cluster had particularly high expression of the neuropeptide CRF and is a population of cells that is known to drive drinking during alcohol dependence [14]. This cluster of cells also expressed the neuropeptides *Tac3, Sst, Nts, and Pdyn*, which have been previously detected in CeA-CRF neurons [4, 36].

Gene expression in other clusters did not obviously correspond to known CeA cell types. For example, two clusters (clusters 4 and 5) expressed elevated levels of *Sst* and *Crhr1*, which are known to colocalize in SST-releasing neurons [1, 39]. SST-releasing cells have been difficult to classify because the SST protein is expressed broadly throughout the CeA, and SST cells tend to co-express other neurotransmitters such as neurotensin, tachykinins, and sometimes CRF [4, 38]. In the current study, these putative SST cells did not highly express any known markers of a specific CeA cell-type but did have high expression of sex hormone receptors.

We identified six clusters of cells that are not known to correspond to CeA neurons and may represent incidental contamination from the adjacent BLA. For example, one cluster (cluster 13) corresponded to cholinergic neurons (Fig. 3A, C). It should be noted that these neurons were only identified during GABAergic subclustering and were not found in the original overall clustering described above. A distinct cluster of intercalated cells was identified (cluster 1, Fig. 3C). Intercalated cells form anatomically distinct cell masses adjacent to the CeA and would likely have been included in our micropunch samples. They were identified by the expression of *Oprm1, Foxp2, Erbb4*, and *Tshz1* [35, 37]. A cluster of putative BLA interneurons (cluster 8) expressing interneuron marker genes, including *Vip, Cck, and Calb2*, and were labeled as VIP interneurons. Cluster 6 highly expressed the serotonin receptor *Htr2a*, which is expressed in the CRF cells of the CeA [1], but these cells did not express any other markers of CeA-CRF neurons. These cells did, however, express the interneuron markers *Kit* and *Lhx6* [40], and were thus identified as *Htr2a* interneurons. Another cluster (cluster 9) was difficult to classify since no obvious CeA markers were expressed. Adarb2 (Adenosine Deaminase RNA Specific B2) was one of the top marker genes of this cluster and thus we identified it as the *Adarb2* interneuron cluster (Fig. 3C). Adarb2 is expressed in a subportion of cortical interneurons and so this cluster may be affiliated with BLA. Additionally, two small clusters were not readily identifiable (cluster 10 and 11). Clusters 10 and 11 contained high levels of glial genes, and formed noncontiguous subclusters in the UMAP plot (Fig. 3A, C), which may indicate that these are GABAergic cells that are contaminated with high levels of glial RNA. These clusters were identified as unknown cells.

### Cell-type specific differential expression in subpopulations of GABAergic neurons

**Fig. 4:**
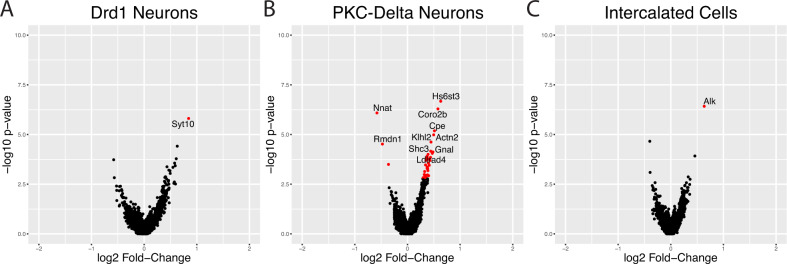
Acute alcohol withdrawal is associated with differential gene expression in specific subtypes of GABAergic neurons in the CeA. Volcano plots are shown for the three subtypes of GABAergic neurons with the greatest numbers of differentially expressed genes (Drd1 Neurons **A**, PKCδ neurons **B**, and intercalated cells **C**). PKCδ cells have by far the greatest number of differentially expressed genes. Genes in red indicate differential expression based on adjusted *p* values of <0.05, and a log2 FC of >±0.25. The top ten differentially expressed genes are labeled.

To determine gene expression changes that were associated with alcohol withdrawal, we performed differential gene expression analysis on each subpopulation of GABAergic neurons to determine the impact of alcohol withdrawal on genes within the cell clusters. DEGs (adjusted *p* value < 0.05, Log2 fold change > ±0.25) were only identified in 3 of the 13 subtypes of GABAergic neurons (Fig. 4A–C*)*. Interestingly, *Prkcd* expressing neurons had the largest number of DEGs (40; 37 upregulated, 3 downregulated) compared with all other cell types, suggesting that this population of neurons is particularly sensitive to alcohol withdrawal. Intercalated cells and *Drd1* expressing cells had only 1 significant DEG each (Fig. 4A, C, respectively). PANTHER gene-ontology analysis [41] revealed that the DEGs in the PKCδ cluster were overrepresented (*p* < 0.05) with glutamatergic signaling genes. Bioplanet analysis revealed 55 pathways that were overrepresented. Notably, this list included a pathway entitled “theoretical pathway for addiction”, with 3 genes present (*Gria1, Camk4*, and *Gria3*).

### Novel marker genes colocalize with PKCδ in the CeA

**Fig. 5:**
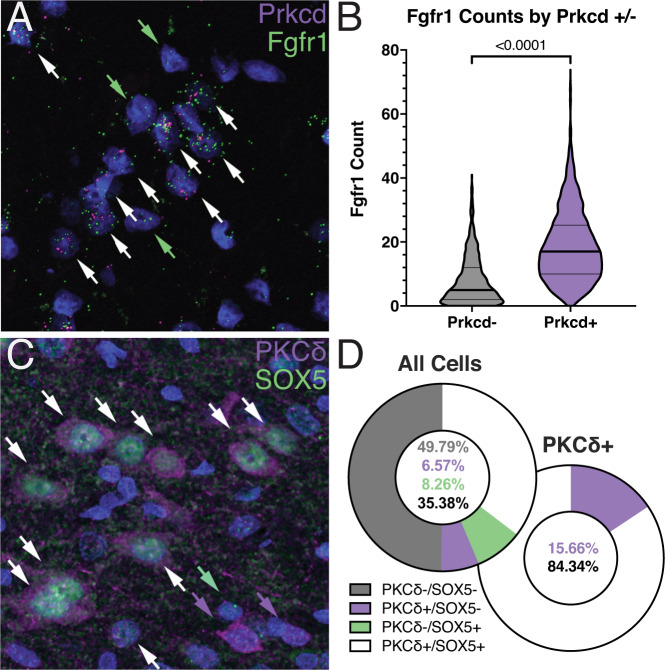
Marker gene validation with RNA in situ hybridization and immunohistochemistry. **A** RNAscope image with *Prkcd* (magenta) and *Fgfr1* (green) puncta and DAPI stained nuclei (blue). Double-labeled nuclei are shown with a white arrow. Twelve total cells are labeled, 9/12 coexpressing *Prkcd* and *Fgfr1* and 3/12 only expressing *Fgfr1*. All *Prkcd*+ nuclei expressed *Fgfr1*. **B** Violin plot of *Fgfr1* expression in *Prkcd*+ (*n* = 542) and *Prkcd*− (*n* = 833) cells from CIE and air exposed animals. Cells that contained ≥3 *Prkcd* puncta were defined as *Prkcd*+. *Prkcd*+ cells contained significantly more *Fgfr1* puncta than *Prkcd*− cells (median = 17 vs. median = 5, *p* < 0.0001 by two-tailed Mann–Whitney test). Notably, zero *Prkcd*+ nuclei (0/542) contained no *Fgfr1* puncta. Animals in this experiment were either exposed to CIE vapor (*n* = 4) or air (*n* = 4) as previously described. **C** Immunofluorescent image with PKCδ (magenta), SOX5 (green) and DAPI (blue) shown. Thirteen total cells are labeled, 10/13 coexpressing PKCδ and SOX5, 2/13 expressing only PKCδ, and 1/13 expressing only SOX5. SOX5 colocalizes with nuclei, while PKCδ is present in the nucleus and cell body. **D** Donut charts of PKCδ and SOX5 coexpression in all cells (*n* = 1659) and in PKCδ expressing cells (*n* = 696). Cells were quantified from 3 sections of CeA tissue (6 bilateral CeA regions) from each of 2 ethanol-naive rats. Images are pseudocolored and adjusted for visibility.

Our data suggested that PKCδ cells in the CeA represent a subpopulation of GABAergic neurons that are particularly sensitive to alcohol withdrawal. snRNA-seq analysis showed that the fibroblast growth factor receptor 1 gene (*Fgfr1)* was abundantly and specifically expressed in PKCδ neurons. To verify that *Fgfr1* mRNA colocalizes with *Prkcd* mRNA in the CeA, we used fluorescent in situ hybridization with RNAScope. A subset of cells in coronal sections of the CeA showed strong *Prkcd* expression (Fig. 5A) with 542/1375 observed cells having >2 *Prkcd* puncta present. These cells were identified and *Prkcd*+ *cells* (Fig. 5A). *Fgfr1* mRNA was also abundant in the CeA (Fig. 5B) and showed substantial overlap with *Prkcd* (Fig. 5C). On average *Prkcd*+ cells expressed over twofold higher more Fgfr1 mRNA than did Prkcd− cells (Fig. 5D, mean 18.7 ± 0.47 versus 7.8 ± 0.25 puncta, respectively, *p* = <0.0001). All 542 Prkcd+ cells expressed contained Fgfr1 puncta. which is consistent with the snRNA-seq analysis. Additionally, we used immunofluorescence to examine colocalization of a second gene product, the protein SOX5 (Sox5) with PKCδ (Fig. 4C). In the snRNA dataset, Sox5 was the top marker of the PKCδ cluster when compared to other GABAergic neurons (expressed in 77.8% of PKCδ cluster neurons, and 21.9% of all other GABAergic neurons). We found that Sox5 was expressed in 84.32% of PKCδ neurons, but in 14.22% of all other cells, indicating that the coexpression found in our dataset is also true at the protein level (Fig. 4D). Both Fgfr1 and SOX5 were present in the lateral portions of the CeA, overlapping spatially with *Prkcd*+ neurons (Fig. S2).

## Discussion

Recent advances in cell-type-specific RNA sequencing technologies allows whole transcriptome profiling from individual cells. Thus, quantitative measures of altered gene expression resulting from disease or drug perturbation can be obtained. These approaches also provide a new index, in the form of novel marker genes, that allows identification and targeting of individual classes of cells based on their unique transcriptional profiles. Until recently, cell specificity relied heavily on histological methods, which can only target a few populations at a time. We used snRNA-seq to provide a cell-type-specific characterization of rat CeA and the transcriptome changes associated with withdrawal induced by intermittent alcohol vapor exposure. Importantly, we identified a subpopulation of CeA GABAergic neurons, PKCδ neurons, that are particularly sensitive to alcohol. The identity of these cells was verified using RNAscope and immunohistochemistry, which confirmed that these neurons were the same PKCδ neurons that had been previously identified in the CeA, but were not previously known to be involved in alcohol dependence or withdrawal.

Some CeA neurons are known to drive escalated alcohol consumption in alcohol dependent and binge-drinking animals [14–16]. As expected, GABAergic cells were the most numerous cell-type detected in the CeA. Subclustering this population of GABAergic neurons allowed us to isolate small populations of cells that had initially been grouped with GABAergic neurons, but were identified as non-GABAergic upon further inspection. These clusters represented a small population of cholinergic neurons, and populations of contaminated cells with unusual glial gene expression that were isolated from the actual GABAergic neurons (Fig. 3A, C). The fact that these cells were detected by subclustering, and not in the initial clustering analysis demonstrates that the refined subclustering analysis can account for small populations of off-target cell types.

Previous studies have used immunohistochemistry and in situ hybridization to identify subpopulations of GABAergic neurons in the CeA. Several cell types including Drd1/ Pdyn, Drd2/Calcrl, PKCδ, SST, and CRF neurons identified in these studies were also identified in our study. Mccullough et al. identified 7 non-overlapping populations of cells in mouse CeA based on the expression of 6 genes (*Prkcd*, *Drd2*, *Sst*, *Tac2*, *Crh*, and *Nts*) [4]. Kim et al. identified 8 populations of CeA neurons, by analyzing the same marker genes and the location of marked populations within the CeA [36]. In our GABAergic subclustering data, we identified 6 populations that are likely CeA cells (although one may be somewhat ambiguous) and that correspond to the populations identified in these previous studies. The first population of known CeA GABA cells that we identified were the PKCδ neurons. PKCδ neurons formed a large population in each of these studies as well as in our data. *Drd2* expressing neurons were the second known population of CeA neurons that we identified (*Drd2*+ neurons, Fig. 3A, C). We found a third large population of neurons that co-express *Drd1, Pdyn, and Tac1*. These cells represent the *Drd1* expressing neurons of the CeA (*Drd1*+ neurons, Fig. 3A, C). In our UMAP plots, *Drd1*+ neurons formed one large cluster, with a smaller noncontiguous cluster near the PKCδ cluster. This may indicate that *Drd1* is expressed in heterogeneous populations of GABA neurons, a conclusion that is supported by studies of mouse CeA [4, 36, 42]. Another major CeA population that we identified expresses the neuropeptide CRF. CRF neurons of the central lateral amygdala are another important population of cells in the CeA that affect fear learning [5, 9, 43–45], and alcohol consumption [14].

Interestingly, some genes that have previously been used to identify CeA neurons were not unique to any specific clusters of cells. For example, *Nts* expressing neurons did not form a distinct cluster, but were instead present in multiple other clusters. This finding is consistent with other studies of the CeA, where *Nts* was detected in *Pdyn*, *Crh*, and *Sst* expressing neurons [15]. Distinct populations of *Sst* expressing cells, which are known to affect fear and stress responses [8, 9, 36, 43] were difficult to identify, because of broad *Sst* expression. However, two clusters of neurons with relatively high *Sst* expression also expressed *Crhr1*, which is specific to *Sst* and *Penk*/*Drd2* neurons [1, 39]. Therefore, we identified them as *Sst* expressing cells (SST neurons 1 and 2, Fig. 3).

We identified three major cell populations in the CeA (GABAergic neurons, astrocytes, and excitatory neurons) with significant DEGs during alcohol withdrawal. In a recent snRNA-seq study from prefrontal cortex of alcohol-dependent humans, glial cells (astrocytes and microglia) were particularly sensitive to the effects of alcohol [27]. Astrocytes had a substantially greater number of DEGs compared with other cell types including glutamatergic and GABAergic neurons. In contrast, in our study using a rat model of dependence we found that, in CeA, GABAergic neurons and astrocytes had similar numbers of DEGs. This suggests that the cell-specific transcriptional response to alcohol dependence and withdrawal are substantially different in CeA and cortex [27], possibly because of the GABAergic composition of the CeA. These findings are consistent with those from mice treated with chronic intermittent alcohol where immune-related glial genes were changed in prefrontal cortex and GABAergic genes were changed in CeA [23]. A highly upregulated gene in GABAergic neurons, *Ubash3b*, was also the top gene that was upregulated by cocaine exposure in rat striatal DRD2 neurons in another study [29]. Expression of the growth factor gene *Fgf2* was upregulated in astrocytes, which is interesting, because the blockade of primary FGF2 receptor, FGFR1, decreases alcohol consumption in rats [46, 47]. Genes that are associated with glutamatergic signaling were differentially regulated in astrocytes and GABAergic neurons. For example, in astrocytes, we found that expression of the cystine-glutamate cotransporter *Slc7a11* was reduced in alcohol withdrawal, which has been previously shown in rat nucleus accumbens [48].

It was surprising to find that *Prkcd* expressing neurons had the greatest number of DEGs during alcohol withdrawal. This finding is novel. Although several studies have focused on the impact of CRF and dynorphin signaling on alcohol consumption and dependence [49], few studies have examined the role of PKCδ neurons in alcohol-related phenotypes. One study linked PKCδ neurons with punishment resistant alcohol drinking, a model of pathological alcohol consumption [17]. Another recent study showed that a subpopulation of PKCδ neurons that expresses *Cartpt* drive drinking in yohimbine stressed animals [19]. PKCδ neurons are associated with negatively valenced stimuli and fear learning [5, 50]. They consistently respond to aversive events and assign a negative valence to conditioned stimuli in a fear learning procedure [5]. This may indicate that PKCδ neurons drive negative emotional states associated with withdrawal, and that they may support negatively reinforcing aspects of alcohol dependence. Further investigation is necessary to uncover the roles of these neurons in specific aspects of alcohol dependence, and targeting them may provide new strategies to study and treat AUD.

Pathway analysis of DEGs in PKCδ neurons revealed several genes involved in glutamatergic signaling and growth factor activation. Upregulated glutamatergic signaling genes included *Gria1*, *Grm3, Ptprd, Ntrk2*, among others. Another upregulated gene, *Cpe*, is a carboxypeptidase that is necessary for the production of mature neuropeptides released by PKCδ neurons, including the endogenous opioid enkephalin [51]. PKCδ neuron marker genes may provide clues about the interactions among alcohol sensitive growth factor genes. For example, we found that the growth factor receptor FGFR1 appears to be a novel marker for the PKCδ cluster. As mentioned above, we previously found that transcripts encoding the FGFR1 ligand FGF2 are increased in astrocytes during alcohol withdrawal. FGF2/FGFR1 signaling has been implicated in excessive alcohol consumption [46, 47] and thus could be a mechanism by which alcohol withdrawal activates PKCδ neurons. FGFR1 signaling could also underlie changes in glutamatergic gene expression in these neurons since FGFR1 activation increases AMPAR insertion into synaptic membranes through a PKC dependent mechanism in the hippocampus [52].

In conclusion, this study used snRNA-seq to characterize cellular transcriptomes of the rat CeA and their response to acute alcohol withdrawal. We identified major cell types in the CeA and subclassified CeA GABAergic neurons. These experiments validated the presence of several previously studied populations of GABAergic neurons in the CeA and were able to identify novel genes specifically expressed in these populations. By using differential expression analysis we found that PKCδ neurons were particularly sensitive to alcohol withdrawal, suggesting that these cells play an important role in alcohol dependence. These findings warrant further research into the role of CeA PKCδ neurons in alcohol withdrawal, alcohol drinking, and negative reinforcement.

## Materials and methods

### Experimental animals

All experimental procedures were approved by the University of Texas at Austin Institutional Animal Care and Use Committee. We used male outbred Wistar rats (Envigo, HSD:Wi) for all experiments. We chose to use male animals for this experiment because it enabled us to pick a withdrawal timepoint that has been shown to affect ethanol consumption when animals show signs of withdrawal, which is a phenotype that has not been extensively studied in females. Animals were 10–12 weeks old at the beginning of ethanol vapor exposure, and 14–16 weeks old at the end of the procedures. Animals were group-housed (3 rats to a cage). Housing rooms maintained a 12-h light, 12-h dark reverse light cycle (lights on at 7:00 p.m. and off at 7:00 a.m.), with ad libitum food access for the duration of the experiment. Cages, food, and bedding were changed twice weekly by the experimenters.

### Ethanol vapor exposure

Rats were exposed to chronic intermittent ethanol (CIE) vapor in custom-made vapor chambers (6 rats per chamber). As previously described [53, 54], vapor-exposed rats were exposed to cycles of ethanol vapor that lasted 14 h a day, and to air for 10 h a day. CIE animals were exposed to ethanol vapor for 4 weeks, or 28 daily sessions. Air exposed control rats lived in a vapor chamber identical to vapor-exposed rats but were not exposed to ethanol vapor. Animals were randomly assigned by cage to either a vapor or control condition.Withdrawal testing and tissue collection occurred 9–10 h after the end of ethanol vapor exposure, in the absence of ethanol vapor. Blood ethanol concentrations were measured weekly using an Analox AM1 Alcohol Analyzer. The target blood ethanol concentration during the fourth week of vapor exposure was 250 mg/dl.

### Somatic signs of withdrawal

Somatic signs of withdrawal were scored using 5 signs, irritability (vocalizations or biting), ventromedial limb retraction, tail stiffness, abnormal gait, and tremors [14, 55]. During withdrawal, animals were briefly removed from their home cage and placed on to a flat surface and observed for signs of withdrawal for 30 s. In order to examine irritability and limb retraction, each animal was briefly lifted by the scruff of the neck at the end of this 30-s period. Each measure was scored on a scale of 0–2 where a 0 indicated that a sign was absent, 1 indicated that the sign was present, and 2 indicated that the sign was severe. One investigator was blinded while another selected rats and recorded scores. Scores from each measure were summed to give an overall withdrawal score.

### Central amygdala tissue collection

CIE and control rats were sacrificed 10 h following the final ethanol vapor exposure. Rat brains were extracted and sliced immediately using a custom-made rat brain slicer. Punches 2 millimeters in diameter were collected from a 2-millimeter-thick slice of brain containing the central amygdala (corresponding to bregma −1.5 mm through bregma −3.5 mm [56]. After collection, tissue punches were immediately frozen on dry ice and stored at −80 °C until nuclei extraction.

### Isolation of nuclei from central amygdala tissue punches

Single-nuclei samples were obtained from CeA tissue punches from 7 male rats (4 CIE exposed rats and 3 Air controls). Each sample consisted of bilateral CeA tissue from a single rat. Nuclei were isolated from frozen tissue punches using iodixanol density gradient centrifugation. CeA tissue punches were homogenized in ice-cold nuclei-sparing lysis buffer (Nuclei EZ Lysis Buffer, Sigma-Aldrich, NUC101-1KT) with 0.2 U/ul RNase inhibitor (NEB, ML314L) and 1X protease inhibitor (Sigma, 4,693,132,001) using a 2 milliliter dounce homogenizer (DWK Life Sciences, 8853000002). Once no visible chunks of tissue remained, the homogenate was digested in the lysis buffer for 5 min and was then strained through a 35-µm cell-strainer (Corning, 352235). The homogenate was then centrifuged at 4 °C at 500 × *g* for 5 min. The pellet was resuspended in a 25% iodixanol solution, which was prepared by combining 583 µL resuspension buffer (PBS with 2% BSA and 0.2 U/µL RNase inhibitor) and 417 µL Optiprep density gradient medium (Sigma-Aldrich, D1556). This sample was then layered on top of a 29% iodixanol solution (569 µL resuspension buffer and 531 µL Optiprep medium) and centrifuged at 13,000 × *g* and 4 °C for 30 min. After density gradient centrifugation, the iodixanol supernatant was removed, and the pellet containing the nuclei was resuspended in 100 µL of resuspension buffer. Nuclei count was obtained using Countess II cell counter (ThermoFisher). To pass quality control, samples were required to have ≥400 nuclei/µL and ≥75% DAPI positive particles. Each sample yielded 500–1700 nuclei/µL of isolate, for a total of 50,000–170,000 nuclei per sample.

### Single-nuclei RNA library preparation and sequencing

Library preparation was performed with a suspension containing an estimated 10,000 nuclei, as measured with a Countess II cell counter. Single-nuclei libraries were prepared using 10X Genomics’ Chromium controller and 3′ single-cell gene expression protocol. Paired-end sequencing was conducted on a NOVASEQ 6000 sequencer and an S2 chip (100 cycles).

### Alignment to reference genome

After nuclei isolation and sequencing, raw sequencing data were analyzed using 10x genomics Cell Ranger software package to map reads to cells and identify the number of cells in each sample. Sequencing data were aligned to the rat genome (Ensemble, Rnor_6.0) using 10X Genomics’ Cell Ranger 5.0 analysis pipeline. A custom rat reference file was created using Cell Ranger’s mkref command, based on the Rnor_6.0 GTF and FASTA files. Then the snRNA Fastq files were aligned to the genome using the Cell Ranger “count” function. Cell Ranger “count” was run with the “include-introns” option active so that unspliced RNAs that are abundant in the nucleus could be mapped to the genome. Cell Ranger “count” produced a gene expression matrix for each sample. We identified 7593–11,525 nuclei in each sample, with 51,090–62,653 reads per nuclei. Cell Ranger detected 7593–11,525 nuclei per sample, with 51,090–62,653 reads per nuclei, and 1809–2516 median genes detected per cell.

### Cluster analysis and identification

Clustering analysis and visualization were performed using Seurat v3.2.1 [57–60]. Prior to clustering analysis, we removed cells with more than 5% mitochondrial reads, cells with less than 200 unique molecular identifiers (UMIs), and likely doublets (beads containing two cells) from the data. All mitochondrial genes and reads were removed from the counts matrix to aid clustering analysis. After quality control 58,640 cells remained (25,941 control and 32,699 ethanol-exposed). Data were normalized and combined into a single object for clustering analysis using the Seurat scTransform package [61].

Prior to clustering, additional cells that did not cluster distinctly and had a high likelihood of being doublets were removed from the data (Scrublet >0.2). Clustering and dimensional reduction analysis were performed using Louvain clustering analysis (resolution = 0.1 for all cells and for GABA neurons) and uniform manifold approximation and projection (UMAP) [62] using 30 principal components. Clusters were identified by marker gene analysis, where marker genes for a known population of cells were examined and compared with the newly identified marker genes in each cluster. In order to determine which genes were the most distinctive, the cells in each cluster were compared with all other cells using the Seurat FindAllMarkers function. The marker genes that were used to identify cells came from a variety of studies that incorporated rat, mouse, and human data. Two clusters were not identifiable in this way and were identified purely based on uniquely expressed marker genes that were identified by Seurat FindAllMarkers.

### Differential gene expression analysis

We used a “pseudobulking” approach to compare cell-type specific gene expression between alcohol exposed and control animals. The raw RNA counts of each gene from all cells in each cluster were added together to produce a pseudobulk gene expression matrix. Then gene expression from alcohol and control animals was normalized and compared using DESEQ2 with batch as a covariate. Genes were considered differentially expressed if they had an adjusted *p* value <0.05 and log2 fold change > 0.25. Gene-ontology analysis was performed using PANTHER v16.0 (geneontology.org [41]).

### Fluorescent in situ hybridization and quantification

We used RNAScope® to quantify *Fgfr1* transcripts in PKCδ positive neurons in the CeA. RNAScope® was performed using manufacturer protocols (ACD Bio). Rats were exposed to CIE ethanol vapor or air as described above, and sacrificed 10 h after the final ethanol vapor session (4 CIE rats and 4 Control rats). Immediately after sacrifice, brains were frozen on isopentane and dry ice. Sections (14 µm) of tissue containing CeA were stained with RNAscope® for *Prkcd*, *Fgfr1*, and nuclei were counterstained with DAPI. Reagents used included probes against rat *Fgfr1* (Rn-Fgfr1-C3, Catalog No. 426958-C3) and *Prkcd* (Rn-Prkcd-C2, Catalog No. 573261-C2). The assay was performed using the RNAscope® Fluorescent Multiplex Reagent Kit (Cat. No. 320850). Slides were cover-slipped with DAPI Fluoromount-G (Southern Biotech, 0100-20) and imaged on a Nikon A1R laser scanning confocal system equipped with a LUN-V 6-line laser unit (405, 488, 561, 640 nm) and a Plan Fluor DIC 40x (Oil) objective (NA—1.3; WD—0.24 mm).

### Immunofluorescence

Wild-type, ethanol-naive rats (*n* = 2) were heavily anesthetized with isoflurane and perfused with phosphate-buffered saline (PBS) followed by 4% paraformaldehyde (PFA) in PBS. Brains were extracted and placed in 4% PFA/PBS at 4 °C overnight, and then transferred to a 30% sucrose solution in PBS at 4 °C. Brains were then sectioned at 20 μm on a cryostat.

Prior to immunostaining CeA sections were washed in PBS and incubated in blocking solution made of 0.2% Triton-X and 3% donkey serum for 1 h at room temperature. Next, sections were incubated overnight at 4 °C with the following primary antibodies in blocking solution: mouse anti-PKCδ (1:1000; 610398, RRID: AB_397781, BD Biosciences) and rabbit anti-SOX5 (1:500; ab94396, Abcam). The next day, sections were washed in PBS and then incubated with the following secondary antibodies in blocking solution for 2 h at room temperature: donkey polyclonal anti-mouse Alexa Fluor 488 (1:500) and donkey polyclonal anti-rabbit Alexa Fluor 594 (1:500). Sections were mounted and cover slipped with DAPI Fluoromount-G (Invitrogen, Cat no. 00-4959-52). Stained tissue was imaged on a Nikon A1R confocal microscope at ×40 magnification. Colocalization of PKCδ with SOX-5 was counted manually using Cell Counter in Fiji after background subtraction.

## Supplementary information

Supplemental Table S1

Supplemental Table S2

Supplemental Table S3

Supplemental Table S4

Supplemental Table S5

Supplemental Table S6

Supplemental Table S7

Supplemental Figure S1

Supplemental Figure S2

Supplemental Figure Legends

## Data Availability

These data are available through the National Center for Biotechnology Information at Bioproject ID: PRJNA796435.

## Code availability

The code used to perform these analyses is available from the investigators upon request.
