# Supplementary material for: Cell-type specific changes in PKC-delta neurons of the central amygdala during alcohol withdrawal
Source: Transl Psychiatry. 2022 Jul 20;12:289. doi: 10.1038/s41398-022-02063-0 (PMC9300707; doi:10.1038/s41398-022-02063-0)
Supplement: Supplementary file 10 — Supplemental Figure Legends [file 41398_2022_2063_MOESM10_ESM.docx]

**Figure S1: Samples and conditions are well represented among each cluster.** Bar plots show the relative proportions of each sample (A) or condition (B) in each cluster. The dotted line in B represents the expected proportion, based on the number of samples in each condition (~0.429; 4 alcohol animals, 3 control animals). **Figure S2: Novel marker genes colocalize with Prkcd.** Left panel: Fluorescent in-situ hybridization (FISH, RNAScope) image of Fgfr1 (green) and Prkcd (magenta). These genes are highly colocalized in the lateral portions of the central amygdala. Right panel : Immunofluorescent image of PKCδ (magenta) and SOX5 (green). SOX5 protein colocalizes with Prkcd in the CeC and CeL. Apparent differences in background are due to the different staining methods used. Abbreviations: BLA: basolateral amygdala; CeM: medial central amygdala; CeL: lateral central amygdala; CeC: capsular central amygdala.
